# A Controlled Trial of Mass Drug Administration to Interrupt Transmission of Multidrug-Resistant Falciparum Malaria in Cambodian Villages

**DOI:** 10.1093/cid/ciy196

**Published:** 2018-03-07

**Authors:** Rupam Tripura, Thomas J Peto, Nguon Chea, Davoeung Chan, Mavuto Mukaka, Pasathorn Sirithiranont, Mehul Dhorda, Cholrawee Promnarate, Mallika Imwong, Lorenz von Seidlein, Jureeporn Duanguppama, Krittaya Patumrat, Rekol Huy, Martin P Grobusch, Nicholas P J Day, Nicholas J White, Arjen M Dondorp

**Affiliations:** 1 Mahidol Oxford Tropical Medicine Research Unit, Faculty of Tropical Medicine, Mahidol University, Bangkok, Thailand; 2 Centre for Tropical Medicine and Global Health, Nuffield Department of Clinical Medicine, University of Oxford, United Kingdom; 3 Center of Tropical Medicine and Travel Medicine, Department of Infectious Diseases, Academic Medical Center, University of Amsterdam, The Netherlands; 4 National Center for Parasitology, Entomology and Malaria Control, Phnom Penh; 5 Provincial Health Department, Battambang, Cambodia; 6 World-Wide Antimalarial Resistance Network, Faculty of Tropical Medicine, Mahidol University, Bangkok, Thailand; 7 Department of Molecular Tropical Medicine and Genetics, Faculty of Tropical Medicine, Mahidol University, Bangkok, Thailand

**Keywords:** subclinical malaria, dihydroartemisinin-piperaquine, malaria elimination, mass drug administration, Southeast Asia

## Abstract

**Background:**

The increase in multidrug-resistant *Plasmodium falciparum* in Southeast Asia suggests a need for acceleration of malaria elimination. We evaluated the effectiveness and safety of mass drug administration (MDA) to interrupt malaria transmission.

**Methods:**

Four malaria-endemic villages in western Cambodia were randomized to 3 rounds of MDA (a 3-day course of dihydroartemisinin with piperaquine-phosphate), administered either early in or at the end of the study period. Comprehensive malaria treatment records were collected during 2014–2017. Subclinical parasite prevalence was estimated by ultrasensitive quantitative polymerase chain reaction quarterly over 12 months.

**Results:**

MDA coverage with at least 1 complete round was 88% (1999/2268), ≥2 rounds 73% (1645/2268), and all 3 rounds 58% (1310/2268). *Plasmodium falciparum* incidence in intervention and control villages was similar over the 12 months prior to the study: 39 per 1000 person-years (PY) vs 45 per 1000 PY (*P* = .50). The primary outcome, *P. falciparum* incidence in the 12 months after MDA, was lower in intervention villages (1.5/1000 PY vs 37.1/1000 PY; incidence rate ratio, 24.5 [95% confidence interval], 3.4–177; *P* = .002). Following MDA in 2016, there were no clinical falciparum malaria cases over 12 months (0/2044 PY) in all 4 villages. *Plasmodium vivax* prevalence decreased markedly in intervention villages following MDA but returned to approximately half the baseline prevalence by 12 months. No severe adverse events were attributed to treatment.

**Conclusions:**

Mass drug administrations achieved high coverage, were safe, and associated with the absence of clinical *P. falciparum* cases for at least 1 year.

**Clinical Trials Registration:**

NCT01872702.

## Teaser

In Cambodian villages, 3-month mass drug administration with high coverage using dihydroartemisinin-piperaquine was safe and was followed by the absence of clinical *Plasmodium falciparum* cases for at least 1 year, despite the presence of multidrug-resistant parasites.

Since 2000, the global malaria burden has declined, making regional malaria elimination and perhaps even eradication more feasible than was thought possible in the last century. To accelerate the decrease in malaria transmission, experts have proposed the implementation of mass drug administrations (MDA). *Plasmodium falciparum* in Southeast Asia has decreased substantially over the last decade, probably related to changes in land use, wide deployment of insecticide-treated bed nets, and, particularly, early diagnosis and treatment of symptomatic disease with artemisinin combination therapies (ACTs). However, partial resistance to artemisinins emerged in western Cambodia in the first decade of the millennium and was subsequently also detected in other regions of the Greater Mekong subregion. The reduced susceptibility of *P. falciparum* to artemisinins is increasingly compounded by ACT partner drug resistance causing high treatment failure rates [1–4]. New antimalarial compounds to replace ACTs will not be available for several years. Preventing the spread of multidrug resistance requires the interruption of local transmission, that is, *P. falciparum* elimination [5–7]. The success of malaria elimination will be accelerated by the treatment of subclinical, low-density parasitemias thought to sustain transmission [8]. Such asymptomatic infections can be detected by highly sensitive screening methods, or presumptively treated by MDAs, which can provide the added benefit of a posttreatment prophylaxis. Systematic reviews show that MDAs have the potential to reduce malaria transmission in certain settings; however, most studies were not designed to demonstrate impact beyond 6 months [7, 9]. In 2015, the World Health Organization (WHO) recommended MDAs in areas where the threat of multidrug resistance requires accelerated elimination efforts and in areas approaching elimination [10, 11].

Rolling out MDA as a tool for malaria elimination in areas of multidrug-resistant falciparum malaria, such as Cambodia, requires a good understanding of the target populations likely to benefit, strategies to ensure sufficient coverage, and the long-term effectiveness of MDA. This study was undertaken to assess the effectiveness of MDAs using dihydroartemisinin-piperaquine (DHA-PQ) in Cambodia, where treatment failures with this combination therapy have been reported [12]. We report findings from a cluster randomized controlled trial to assess the effectiveness of 3 rounds of MDA using DHA-PQ to interrupt malaria transmission.

## METHODS

**Figure 1.**
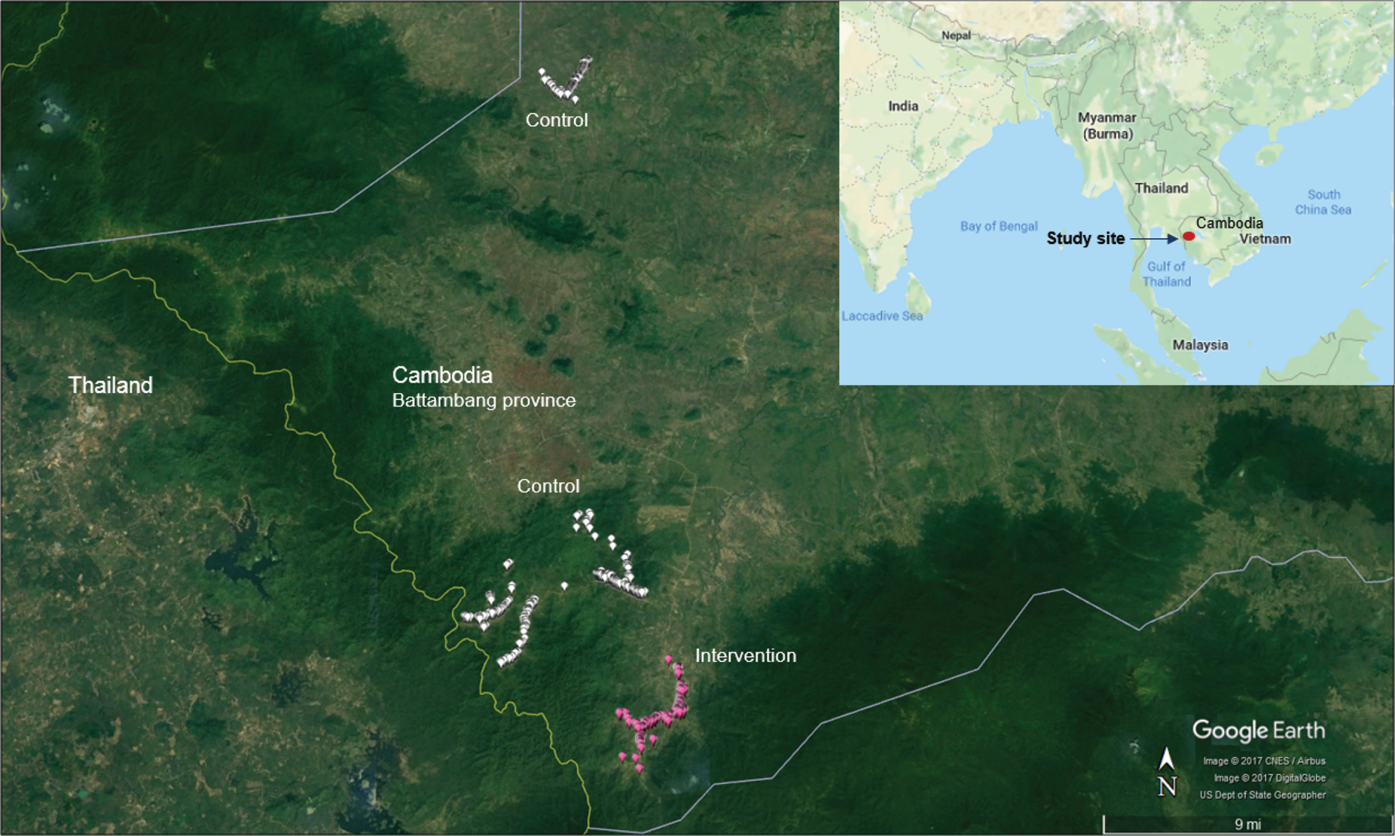
Household-level map of study villages. Each marker represents 1 household. Pink markers indicate households in intervention villages that received mass drug administration during July–September 2015. White markers indicate households in control villages that received mass drug administration during July–September 2016.

Initial surveys in 20 western Cambodian villages showed low prevalences of subclinical *P. falciparum* infections except in villages near forested areas [13]. Four forest-fringe villages along the Thai border were selected for the current study based on *P. falciparum* prevalence and accessibility. The villages were randomized to MDA (intervention) in year 1 or deferred MDA 1 year later (control). Randomization was restricted, with 2 pairs of villages matched for geographical proximity and parasite prevalence (Figure 1). The only form of vector control in the study villages was the use of bed nets. Long-lasting insecticide-treated bed nets (LLITNs) are distributed by the national malaria control program every 3 years. A mass distribution of LLITNs took place in early 2015 prior to the study start. Bed net use was high in the study area as bed nets have been used historically to protect not only against malaria but also against nuisance insects.

### Community Mobilization and Informed Consent

Individual written informed consent was obtained from all participants. A fingerprint was obtained for illiterate participants and countersigned by a witness. Intensive community engagement activities in participating villages have been described elsewhere and included meetings by a dedicated Khmer-speaking community engagement team with commune authorities, village malaria workers (VMWs), village leaders, and other villagers [14–16].

### Mass Drug Administrations

**Figure 2.**
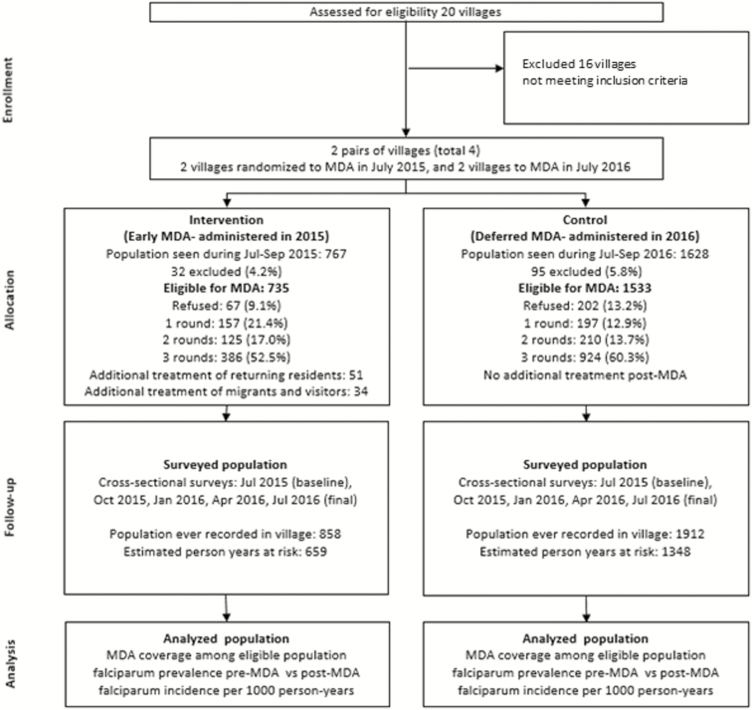
Study flowchart. One additional hamlet from a control village was included in the final cross-sectional survey in July 2016 and then in the subsequent MDA. Abbreviation: MDA, mass drug administration.

For each round of treatment, a mobile clinic was set up in the village scheduled for MDA, but participants could also receive MDA at home. MDA rounds were conducted once a month for 3 consecutive months early in the rainy season. Local health staff administered a directly observed 3-day regimen of 7 mg/kg dihydroartemisinin and 55 mg/kg piperaquine tetraphosphate (Eurartesim, Sigma Tau, Italy) to the entire village population. Newcomers or returning residents arriving during the study period were contacted by VMWs and offered a single round of PQ. Infants <6 months of age, pregnant or lactating women, and anyone with acute health problems were excluded from MDA. Participants were asked about adverse events on days 1, 2, 3, and 7 after drug administration. Any severe illness, hospitalization, or death was investigated by the study team and its association with treatment until 30 days following each drug administration was assessed (Figure 2).

### Quarterly Prevalence Surveys

A cross-sectional survey was conducted every 3 months through July 2015 to July 2016. Participants with fever ≥37.5°C were tested by a rapid diagnostic test (RDT; SD BIOLINE Malaria Ag Pf/Pv, Standard Diagnostics, Gyeonggi-do, Republic of Korea) and treated with standard treatment if positive. Ethylenediaminetetraacetic acid–anticoagulated venous blood was collected on ice and transported in a cool-box to the laboratory on the same day.

### Clinical Malaria Incidence Records

In all villages, all febrile patients were tested for malaria by RDT by an existing VMW, and treated according to national guidelines when indicated. The study team supervised VMWs weekly and collected malaria case records during July 2015 to June 2017. These, along with records going back to January 2014, were matched to study participants, and this information was confirmed during a home visit by the study team. Additionally, between July 2015 to December 2016, VMWs also obtained a dried blood spot stored in plastic bags with silica gel, as well as travel histories from all patients with RDT-confirmed malaria. Malaria incidence data from January 2014 to June 2015 were obtained from the Provincial Health Department.

### Laboratory Methods

An ultrasensitive, quantitative polymerase chain reaction method (uPCR) with a lower limit of detection of 22 parasites/mL of whole blood was used to detect and used to quantify *Plasmodium* densities, as described previously [17]. To detect mutations in the gene on chromosome 13 (*PfKelch13*) associated with artemisinin resistance, the open reading frame of the PF3D7_1343700 kelch propeller domain was amplified using nested PCR [17]. Purified PCR products were sequenced at Macrogen (Republic of Korea) and analyzed using BioEdit version 7.1.3.0, using the 3D7 kelch13 sequence as reference (accession number XM_001350122.1) [17].

### Data Management and Statistical Analysis

Each individual received a unique identification number during a census immediately prior to the initiation of the study or when first seen during the study. Clinical malaria records were linked to cross-sectional surveys and MDA treatment registers by field workers who confirmed the identity and status of participants. Coverage of MDA was estimated from the population present in the village at any time point during the 3-month period during which MDA occurred. Survey data were collected on case record forms and entered on smartphones using ODK software [18] before being exported into OpenClinica [19].

The primary analyses compared the impact on incidence of malaria, defined as confirmed *Plasmodium* infections accompanied by clinical manifestations or the subclinical prevalence of *Plasmodium* infections. The first analysis compared *P. falciparum* prevalence at baseline, 4 months, and 12 months, in villages randomized to either early MDA (intervention) or deferred MDA (control). Changes in the prevalence of *Plasmodium* infections were analyzed using a generalized estimating equation that took clustering into account. In a second analysis, the incidence of *P. falciparum* malaria episodes including mixed vivax/falciparum episodes were compared in the 12-month period before and after MDA. Incidence rate ratios (IRRs) and incidence rate differences were calculated by Poisson regression comparing intervention vs control, and, separately, before and after MDA within each study arm. The denominator for clinical malaria incidence was all residents who could have contributed to the transmission of malaria. Each individual’s time in the village was estimated, irrespective of participation, from the 5 cross-sectional surveys and 3 rounds of MDA and recorded as present or absent in the village. Person-years at risk during prestudy and poststudy periods were adjusted based on natural population growth, assumed to be 1.91% during 2014–2015 and 1.85% during 2016–2017 [20].

The 4 village clusters in Cambodia were part of a larger multicenter study in Southeast Asia and were not powered independently to provide statistically robust findings. Significance values were determined at the 5% level. MDA and treatment data were recorded on registers and then entered in a Microsoft Excel spreadsheet. Analyses were done using Stata version 14.0 software (StataCorp, College Station, Texas).

### Ethical Considerations

Ethical approval was obtained from the Cambodian National Ethics Committee for Health Research (reference numbers 012, 29.01.2014; 042, 20.02.2015; 051 18.02.2016) and the Oxford Tropical Research Ethics Committee (reference number 1017-13). The study was registered at ClinicalTrials.gov (NCT01872702).

## RESULTS

Of those present in the villages during the period of the MDAs, 2268 of 2395 (95%) were eligible for treatment. Coverage with 1 complete round of MDA was 1999 of 2268 (88%) residents, 2 complete rounds in 1645 of 2268 (73%) residents, and 3 complete rounds in 1314 of 2268 (58%) residents. A total of 269 of 2268 (12%) of those present and eligible did not receive any MDA. Of the residents who returned to the village after the MDA intervention, 51 of 72 (71%) received 1 full course of DHA-PQ, as did 34 visitors. Among 127 of 2395 (5%) individuals who were excluded from MDA, 50 (39%) had an acute illness, 43 (34%) were pregnant women, and 25 (20%) were lactating mothers. Ten (1%) residents were excluded for other reasons. In total, 2770 people were recorded as being in the villages during at least 1 of the MDA rounds or during 1 of the cross-sectional surveys (Figure 2). The participants in the intervention and control arm were similar in terms of sex, age, and occupation (Table 1). Of study participants, 91% (2148/2354) stated that they used bed nets regularly. There was no significant difference in bed net use between villages with early and deferred MDA.

**Table 1.** Overall Study Population Characteristics and Drug Administration Status

Characteristic	Total, No.	(%)	Intervention, No.	(%)	Control, No.	(%)
Population	2770		858		1912	
Sex						
Female	1319	(47.6)	376	(43.8)	943	(49.3)
Male	1451	(52.4)	482	(56.2)	969	(50.7)
Age group, y						
<5	280	(10.1)	71	(8.3)	209	(10.9)
5–14	599	(21.6)	140	(16.3)	459	(24.0)
15–44	1326	(47.9)	452	(52.7)	874	(45.7)
≥45	565	(20.4)	195	(22.7)	370	(19.4)
Occupation						
Child or student	1021	(36.9)	274	(31.9)	747	(39.0)
Farmer working around forest	303	(10.9)	34	(4.0)	269	(14.1)
Farmer working around village	1152	(41.6)	417	(48.6)	735	(38.5)
Other working around village	101	(3.7)	38	(4.4)	63	(3.3)
Other working away from village	193	(6.9)	95	(11.1)	98	(5.1)
Residency duration, y						
<1	332	(12.0)	113	(13.2)	219	(11.4)
≥1	2438	(88.0)	745	(86.8)	1693	(88.6)
Residency status						
Resident	2340	(84.5)	773	(90.1)	1567	(82.0)
Nonresident	430	(15.5)	85	(9.9)	345	(18.0)
Bed net use						
Everyday	2148	(91.2)	655	(87.3)	1493	(93.1)
Irregular	206	(8.8)	95	(12.7)	111	(6.9)
Away and excluded						
Away throughout 3 mo of MDA	303	(11.2)	19	(2.4)	284	(14.9)
Excluded	127	(5.3)	32	(4.2)	95	(5.8)
Coverage among population present and eligible during MDA (n = 2268)						
Refused or did not attend MDA	269	(11.9)	67	(9.1)	202	(13.2)
1 round of MDA	354	(15.6)	157	(21.4)	197	(12.9)
2 rounds of MDA	335	(14.8)	125	(17.0)	210	(13.7)
3 rounds of MDA	1310	(57.8)	386	(52.5)	924	(60.3)
At least 1 round of MDA	1999	(88.1)	668	(90.9)	1331	(86.8)
At least 2 rounds of MDA	1645	(72.5)	511	(69.5)	1134	(74.0)
Post-MDA treatment of returning residents (n = 72)						
Received 1 round of treatment	51		51	(70.8)	0	0

Abbreviation: MDA, mass drug administration.

### MDA Effectiveness Against Clinical *Plasmodium* Incidence

**Figure 3.**
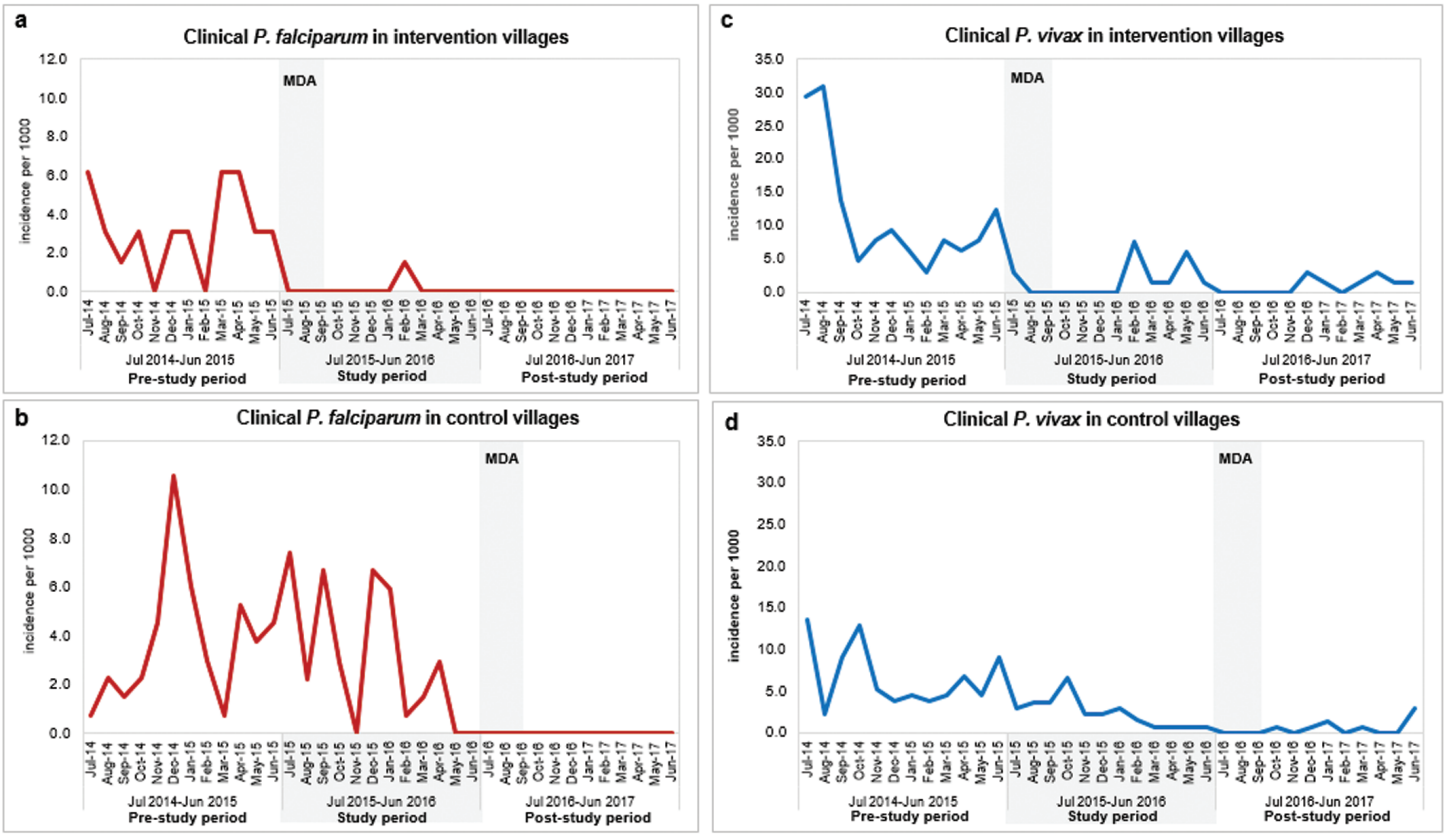
Clinical malaria incidence, July 2014–June 2017: before MDA, during the study, and after cross-over treatment in early MDA (intervention) and delayed MDA (control) villages. *A* and *B*, *Plasmodium falciparum* incidence in intervention and control villages, respectively. *C* and *D*, *Plasmodium vivax* incidence in intervention villages and control villages, respectively. Abbreviation: MDA, mass drug administration.

Over the 12 months following the start of MDA (July 2015 to June 2016), falciparum malaria incidence was fell to 1 in 659 (1.5/1000 per year) in the intervention villages compared with 50 of 1348 (37.1/1000 per year) in control villages that had not yet received MDA (IRR, 24.5 [95% confidence interval {CI}, 3.4–177]; *P* = .002). In the prestudy period (July 2014–June 2015), there was no significant difference (IRR, 1.2 [95% CI, .7–1.9]; *P* = .503) in falciparum malaria incidence between the intervention (25/646 [39/1000 per year]) and control villages (60/1322 [45/1000 per year]). The control villages received MDA at the end of the study period, and clinical cases were monitored during the poststudy period from July 2016 to June 2017. No falciparum malaria cases were reported in any of the 4 study villages during the poststudy period (Figure 3 and Table 2).

**Table 2.** Clinical Malaria Incidence per 1000 Person-years^a^ (Cases/Population at Risk) in Early Mass Drug Administration (MDA) (Intervention) and Deferred MDA (Control) Villages: Before MDA, During the Study, and After Deferred MDA

Period	*Plasmodium falciparum* or Mixed Infections				*Plasmodium vivax*			
	Incidence		IRR (95% CI)	*P* Value	Incidence		IRR (95% CI)	*P* Value
	Intervention	Control			Intervention	Control		
Prestudy period (July 2014–June 2015)	38.7 (25/646)	45.4 (60/1322)	1.2 (.7–1.9)	.503	139.2 (90/646)	80.1 (106/1322)	0.6 (.4–.8)	<.001
Study period (July 2015–June 2016)	1.5 (1/659)	37.1 (50/1348)	24.5 (3.4–177.0)	.002	21.2 (14/659)	28.9 (39/1348)	1.4 (.7–2.5)	.322
Poststudy period (July 2016–June 2017)	0.0 (0/671)	0.0 (0/1373)	1	…	11.9 (8/671)	6.5 (9/1373)	0.5 (.2–1.4)	.218

Abbreviations: CI, confidence interval; IRR, incidence rate ratio.

^a^Person-years calculated as time at risk contributed by the population ever recorded as present in a study village (whether they participated in mass drug administration [MDA] or a survey or not) established at 8 time points (each month of MDA and each of 5 prevalence surveys). Population defined during July 2015 to June 2016, and natural population growth assumed to be 1.91% during 2014–2015 and 1.85% during 2016–2017 [20]. *P* values for IRRs were obtained by Poisson regression.

Within each treatment arm, we also compared the incidence of falciparum malaria during the 12 months before MDA against the 12 months following MDA. In villages that received MDA in 2015, *P. falciparum* incidence was 25 of 646 (39/1000 per year) prior to MDA vs 1 of 659 (1.5/1000 per year) after MDA (IRR, 25.5, [95% CI, 3.5–188]; *P* = .001). In villages that received MDA in 2016, falciparum malaria incidence was 50 of 1348 (37/1000 per year) before MDA vs 0 of 1373 after MDA (Supplementary Table 1). The post-MDA falciparum malaria incidence from July 2016 to June 2017 in all villages was 0 of 2044 person-years. One infection was detected in a forest worker who had not received MDA during the study period from July 2015 to June 2016 (Table 2 and Supplementary Table 1).

Vivax malaria incidence did not differ significantly between intervention (14/659 [21/1000 per year]) and control villages (39/1348 [29/1000 per year]) during the study period from July 2015 to the end of June 2016 (IRR, 1.4 [95% CI, .7–2.5]; *P* = .322; Figure 3 and Table 2). Comparing pre- and post-MDA periods, there was a significant reduction of vivax malaria incidence from 90 of 646 (139/1000 per year) to 14 of 659 (21/1000 per year) in the intervention villages (IRR, 6.6 [95% CI, 3.7–11.5]; *P* < .001) and from 39 of 1348 (29/1000 per year) to 9 of 1373 (6.5/1000) per year in the control villages (IRR, 4.4 [95% CI, 2.1–9.1]; *P* < .001 (Supplementary Table 1).

### MDA Impact on Subclinical *Plasmodium* Infections

**Figure 4.**
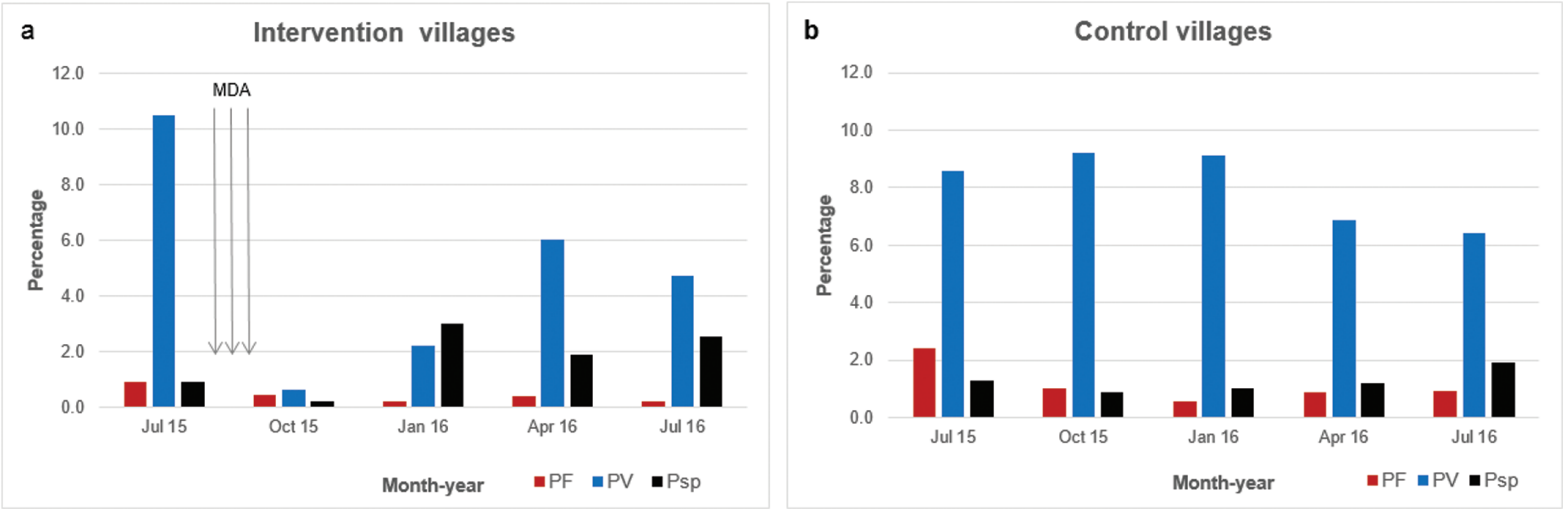
Subclinical *Plasmodium* prevalence detected by quantitative ultrasensitive PCR (uPCR): intervention vs control villages. *A*, *Plasmodium* species prevalence by ultrasensitive PCR (uPCR) in intervention villages following 3 rounds of mass drug administration. *B*, *Plasmodium species* prevalence by uPCR in control villages. Abbreviations: MDA, mass drug administration; PF, *Plasmodium falciparum* or mixed *P. falciparum* infections; Psp, *Plasmodium* infections, unknown species; PV, *Plasmodium vivax* infections.

The prevalence of subclinical *P. falciparum* was low and declined in all villages irrespective of treatment allocation. Comparing baseline (pre-MDA) with 4 months after the start of MDA, the prevalence of subclinical *P. falciparum* infections assessed by uPCR was 5 of 543 (0.9%) vs 2 of 470 (0.4%) in intervention villages, and 17 of 701 (2.4%) vs 7 of 696 (1.0%) in control villages. The odds ratio (OR) was 0.15 (95% CI, .02–1.31; *P* = .09), with no significantly different change against baseline. By 12 months, the prevalence of subclinical *P. falciparum* infections was 1 of 512 (0.2%) in intervention villages, compared with 10 of 1090 (0.9%) in control villages (OR, 0.78 [95% CI, .07–8.84]; *P* = .840; Figure 4 and Table 3). Among 14 people with subclinical *P. falciparum* infections immediately prior to MDA, 13 (93%) became negative throughout the follow-up period. One study participant with a subclinical *P. falciparum* infection before MDA remained infected after receiving 3 rounds of MDA.

**Table 3.** Subclinical Parasite Prevalence by Ultrasensitive, Quantitative Polymerase Chain Reaction: Comparison of Proportional Changes in Early Mass Drug Administration (MDA) (Intervention) Versus Deferred MDA (Control) Villages at Month 4 and Month 12

Period	Intervention				Control				*Plasmodium falciparum* or Mixed Infections		*Plasmodium vivax*	
	Total, No.	Pf or Mixed	PV	*Plasmodium* Species^a^	Total	Pf or Mixed	PV	*Plasmodium* Species	OR (95% CI)	*P* Value	OR (95% CI)	*P* Value
July 2015 (baseline)	543	5 (0.9)	57 (10.5)	5 (0.9)	701	17 (2.4)	60 (8.6)	9 (1.3)				
October 2015	470	2 (0.4)	3 (0.6)	1 (0.2)	696	7 (1.0)	64 (9.2)	6 (0.9)	0.15 (.02–1.31)	.086	0.02 (.003–.13)	<.001
January 2016	504	1 (0.2)	11 (2.2)	15 (3.0)	692	4 (0.6)	63 (9.1)	7 (1.0)				
April 2016	531	2 (0.4)	32 (6.0)	10 (1.9)	583	5 (0.9)	40 (6.9)	7 (1.2)				
July 2016^b^	512	1 (0.2)	24 (4.7)	13 (2.5)	1090^c^	10 (0.9)	70 (6.4)	21 (1.9)	0.78 (.07–8.84)	.840	0.65 (.41–1.05)	.076

Data are presented as No. (%) unless otherwise indicated.

Abbreviations: CI, confidence interval; OR, odds ratio; Pf, *Plasmodium falciparum*; PV, *Plasmodium vivax*.

^a^ *Plasmodium* species identified by ultrasensitive polymerase chain reaction (uPCR), but nested PCR unable to determine which one (chiefly due to limitations of amplification of low amounts of initial genetic material).

^b^Using a generalized estimating equation that took clustering into account, proportional change of subclinical *P. falciparum* prevalence, comparing baseline vs 12 months after: intervention, 5 of 543 (0.9%) vs 1 of 512 (0.2%); control, 17 of 701 (2.4%) vs 10 of 1090 (0.9%); OR, 0.78 (95% CI, .07–8.84); *P* = .840. Proportional change of subclinical *P. falciparum* prevalence, comparing baseline vs 4 months after: intervention, 5 of 543 (0.9%) vs 2 of 470 (0.4%); control, 17 of 701 (2.4%) vs 7 of 696 (1.0%); OR, 0.15 (95% CI, .02–1.31); *P* = .086.

^c^An additional hamlet in PRY village (control) participated in the July 2016 cross-sectional survey.

Subclinical *P. vivax* infections were more prevalent than subclinical *P. falciparum* infections in all surveys. At 4 months, there was a large decline in the prevalence of subclinical *P. vivax* infections in intervention villages, 57 of 543 (10.5%) to 3 of 470 (0.6%), and only a small decline in control villages (60 of 701 [8.6%] to 70 of 696 [6.4%]) (OR, 0.02 [95% CI, .003–.13]; *P* < .001). By 12 months, subclinical *P. vivax* infections in intervention villages had returned to approximately half of the baseline prevalence, and subclinical *P. vivax* infection was no longer significantly lower than in control villages (24 of 512 [4.7%] in intervention villages vs 70 of 1090 [9.2%] in control villages; OR, 0.65 [95% CI, .41–1.05]; *P* = .076; Figure 4 and Table 3).

Of the 57 individuals with uPCR-detected *P. vivax* infections immediately prior to MDA, 24 (42%) remained free of infections throughout the 12-month follow-up period whereas 23 (40%) had subclinical *P. vivax* infections. The status of 10 (18%) participants was unknown as they did not attend subsequent surveys.

### Markers of Antimalarial Drug Resistance

Molecular markers for artemisinin resistance were detected in both subclinical and clinical falciparum malaria episodes. Of 56 *P. falciparum* parasite strains detected by uPCR, 27 (48%) were sequenced for kelch 13 mutations, and all 27 were positive for the C580Y haplotype. None was positive for other K13 markers. Among 35 *falciparum* malaria cases detected up to April 2016, all had a mutation associated with artemisinin resistance in the Kelch 13 gene: 34 had the K13 C580Y mutation and 1 had the K13 F446I mutation.

### Tolerability and Safety of DHA-PQ

In total, 14845 DHA-PQ tablets were administered during MDA, and adverse events were reported by 45.5% (909/1999) of participants who received treatment (Supplementary Table 2). The most common adverse events were dizziness (22% [431/1999]), headache (18% [368/1999]), fever (10% [195/1999]), and nausea (8% [163/1999]). All other symptoms were reported in <5% of participants. Vomiting of study drugs was reported by 2% (33/1999) participants. Of adverse events, 96% (869/909) were mild in nature and no medical treatment was sought; 4.4% (40/909) of the adverse events required medical attention from the study team or a local healthcare provider. During the 4-month passive surveillance periods during and after MDAs in 2015 and 2016, 3 deaths were reported among participants: 1 homicide and 2 cases of accidental drowning. None of the deaths was attributed to the study medications by an independent medical review. No other severe adverse events were reported.

## DISCUSSION

Deployment of 3 rounds of MDA reduced falciparum malaria incidence dramatically and rapidly. A single case of falciparum malaria was detected in the year after MDA, compared to 24 cases in the same villages in the previous year, and 50 cases in untreated villages. No clinical falciparum malaria cases were detected in intervention or control villages in the year after MDA, with the exception of a forest worker who did not participate in the MDA. Forest work is a well-documented risk factor for malaria in the study region [21–26]. Following MDA, the prevalence of *P. falciparum* infections decreased in the study villages but did not reach zero. The subclinical *falciparum* infections did not correlate with clinical malaria incidence, which might be related to the remaining asymptomatic carriers.

Our study employed 3 rounds of DHA-PQ, which facilitates the capture of short-term travelers and the elimination of infections circulating in vectors [27]. A full piperaquine course provides a posttreatment prophylactic effect of around a month [28]. During the study period, treatment failures with DHA-PQ increased in Cambodia [4, 29], prompting a change in first-line treatment to artesunate-mefloquine. In our study, all tested parasites carried the *PfKelch13* marker for artemisinin resistance. All low-density subclinical *P. falciparum* infections but one were cleared by the DHA-PQ regimen. Increasing drug resistance is, however, an important concern for the future use of DHA-PQ for MDA in this area.

*Plasmodium vivax* incidence and prevalence decreased substantially following MDA, but rebounded by 6 months and had returned to approximately half the pre-MDA level 1 year after the MDA. This has been observed also in other MDA trials [30], and is best explained by the hypnozoite stage of *P. vivax* causing relapses of the primary infection. Hypnozoites are not affected by the MDA regimen and require a prolonged course of primaquine.

The MDA was remarkably safe and well accepted. Few severe adverse events and no drug-related severe adverse events were reported. As the study did not include a control group receiving placebo, it is not possible to assess reliably the true adverse event frequency attributable to the study drugs. Factors affecting acceptability have been reported earlier, and confirmed that the perceived side effects of the drug administration were common but mild [31].

MDA was conducted in individual villages surrounded by untreated villages. Malaria can be reintroduced by visitors from neighboring villages or through residents who are exposed when staying outside their village. Forest workers are at high risk to reintroduce *Plasmodium* infections into villages after the interruption of malaria transmission. MDA conducted in geographically distinct villages well connected by roads, as in this study, cannot prevent the reintroduction of falciparum malaria unless a wider region is targeted.

A major limitation of this study is the number of randomized clusters. With only 2 clusters per arm, statistical significance testing is unstable, yet our results still suggest a real difference between study arms and before vs after the intervention. The study is part of a larger multicenter trial in Myanmar, Vietnam, and Lao People’s Democratic Republic. The combined analysis of the studies is expected to have sufficient power to detect statistically significant differences. Another limitation is the absence of an entomological research component that could have helped demonstrate interruption of transmission. Detailed entomological observations were collected in Myanmar, one of the other studies in the project [32]. Finally, for programmatic roll-out and scale-up of MDAs, the cost efficacy and cost- benefit ratios of the intervention are needed. Such economic considerations are difficult in targeted malaria elimination studies where the economic benefits of local malaria elimination have to be speculative.

A similar impact of the same MDA regimen as an addition to establishment of a network of malaria posts has recently been demonstrated in Myanmar [30]. MDA efforts elsewhere have interrupted malaria transmission permanently on Pacific islands [33]. In other regions the impact of MDA was more transient [9, 34]. Parameters that can affect the impact of MDA include the drug regimen, coverage, and local malaria epidemiology [9, 34]. MDA is most likely to be successful in low-transmission settings where high coverage can be achieved [35]. The WHO now supports MDA as an additional tool for *P. falciparum* elimination in low-endemicity regions including Southeast Asia [10, 11, 36]. Several novel approaches to boost the impact of MDAs, including the prevention of reintroduction of *Plasmodium* infections by screening and treating visitors and immigrants, immunization of residents with a malaria vaccine candidate, and reducing vector density by adding ivermectin to MDAs are under discussion.

## CONCLUSIONS

In our preelimination setting, MDA (integrated with appropriate case management, use of LLITNs, and supported by effective community mobilization) resulted in absence of clinical *P. falciparum* cases for at least 1 year. The impact on the prevalence of subclinical *P. vivax* infections was much more limited. Additional interventions including radical cure with primaquine will be needed to eliminate vivax malaria. The careful selection of quality drugs in the most appropriate drug regimens, the targeting of suitable villages, and allocation of adequate resources needed to achieve high coverage will be critical to address during the expansion of MDA programs in Southeast Asia.

## Supplementary Data

Supplementary materials are available at *Clinical Infectious Diseases* online. Consisting of data provided by the authors to benefit the reader, the posted materials are not copyedited and are the sole responsibility of the authors, so questions or comments should be addressed to the corresponding author.

Supplementary Table 1

Supplementary Table 2

Supplementary Table 3

## Notes

***Author contributions.*** R. T. and T. J. P. conducted the study. L. v. S., C. D., and C. N. supervised the study. A. M. D., N. P. D., and N. J. W. conceived of and designed the study. H. R. and M. P. G. provided scientific oversight. C. P., J. D., K. P., M. D., M. I., and P. S. supervised the study samples, molecular laboratory, and data management. R. T., T. J. P., and M. M. analyzed the data. R. T. and T. J. P. wrote the manuscript, and all authors contributed to and approved its final version.

***Acknowledgments.*** We thank the study participants and communities, village malaria workers, health centers, and local authorities. We also thank Im Sambo, Chhouen Heng, Nou Sanann, Pich Kunthea, Moeun Moninh, Ma Sareth, Roeun Sary, Chan Bora, Kem Sovann, Chan Chang, Su Nin, Un Oeun, Buth Sophany, Pengby Ngor, Mark Debackere, Honey Moon, Myo Myo, and Suphak Nosten, Phaikyeong Cheah, Susanna Dunachie, Olivo Miotto, Patrick Hannay, Patchara Kitlapcharoenkul, Richard Maude, Nisarat Sorotpinya, and Jaruwan Tubprasert.

***Financial support.*** This work was supported by the Bill & Melinda Gates Foundation (award number OPP1081420), and was conducted as part of the Wellcome Trust–Mahidol University–Oxford Tropical Medicine Research Programme, which is funded by the Wellcome Trust of Great Britain (award number 101148/Z/13/Z). The study sponsor is University of Oxford, United Kingdom.

***Potential conflicts of interest.*** All authors: No reported conflicts of interest. All authors have submitted the ICMJE Form for Disclosure of Potential Conflicts of Interest. Conflicts that the editors consider relevant to the content of the manuscript have been disclosed.
