# Supplementary material for: A Controlled Trial of Mass Drug Administration to Interrupt Transmission of Multidrug-Resistant Falciparum Malaria in Cambodian Villages
Source: Clin Infect Dis. 2018 Mar 7;67(6):817–26. doi: 10.1093/cid/ciy196 (PMC6117448; doi:10.1093/cid/ciy196)
Supplement: Supplementary Table 1 [file ciy196_suppl_supplementary-table-1.docx]

| **Period** | **Intervention** | | | | | | **Control** | | | | | |
| --- | --- | --- | --- | --- | --- | --- | --- | --- | --- | --- | --- | --- |
|  | ***P.falciparum* or mixed infections** | | | ***P.vivax*** | | | ***P.falciparum* or mixed infections** | | | ***P.vivax*** | | |
|  | **Incidence** | **IRR**  **(95% CI)** | **p-value** | **Incidence** | **IRR**  **(95% CI)** | **p-value** | **Incidence** | **IRR**  **(95% CI)** | **p-value** | **Incidence** | **IRR**  **(95% CI)** | **p-value** |
| **Pre-study period** | 38.7  (25/646) | 25.5  (3.5-188.2) | 0.001 | 139.2  (90/646) | 6.6  (3.7-11.5) | <0.001 | 45.4  (60/1,322) |  |  | 80.1  (106/1,322) |  |  |
| **Study period** | 1.5  (1/659) |  |  | 21.2  (14/659) |  |  | 37.1  (50/1,348) | NA | NA | 28.9  (39/1,348) | 4.4  (2.1-9.1) | <0.001 |
| **Post-study period** | 0.0  (0/671) |  |  | 11.9  (8/671) |  |  | 0.0  (0/1,373) |  |  | 6.5  (9/1,373) |  |  |

**Supplement Table 1: Clinical malaria incidence per 1000 person-years (cases/pop at risk) before vs after MDA in early MDA (intervention) and deferred MDA (control) villages.** Clinical malaria incidence during July 2014 – June 2015 (Pre-study period before mass drug administration), July 2015-June 2016 (Study period after MDA in intervention villages), and July 2016-June 2017 (Post-study period after MDA in control villages). p-values for incidence rate ratios were obtained by Poisson regression
