# Supplementary material for: A Controlled Trial of Mass Drug Administration to Interrupt Transmission of Multidrug-Resistant Falciparum Malaria in Cambodian Villages
Source: Clin Infect Dis. 2018 Mar 7;67(6):817–26. doi: 10.1093/cid/ciy196 (PMC6117448; doi:10.1093/cid/ciy196)
Supplement: Supplementary Table 2 [file ciy196_suppl_supplementary-table-2.docx]

**Supplement Table 2: Adverse events amongst participants who received MDA**

| Adverse Events (AE) | Number of Participants with AE | % | *Severity | | | |
| --- | --- | --- | --- | --- | --- | --- |
|  |  |  | Mild % | | Moderate % | |
| Dizziness | 431 | 21.6 | 423 | 98.1 | 8 | 1.9 |
| Headache | 368 | 18.4 | 348 | 94.6 | 20 | 5.4 |
| Fever | 195 | 9.8 | 177 | 90.8 | 18 | 9.2 |
| Nausea | 163 | 8.2 | 156 | 95.7 | 7 | 4.3 |
| Fatigue | 98 | 4.9 | 89 | 90.8 | 9 | 9.2 |
| Running nose | 94 | 4.7 | 93 | 98.9 | 1 | 1.1 |
| Feeling unwell | 65 | 3.3 | 64 | 98.5 | 1 | 1.5 |
| Diarrhea | 35 | 1.8 | 32 | 91.4 | 3 | 8.6 |
| Vomiting | 33 | 1.7 | 30 | 90.9 | 3 | 9.1 |
| Abdominal pain | 32 | 1.6 | 29 | 90.6 | 3 | 9.4 |
| Palpitation | 25 | 1.3 | 24 | 96.0 | 1 | 4.0 |
| Muscle pain | 24 | 1.2 | 22 | 91.7 | 2 | 8.3 |
| Poor appetite | 12 | 0.6 | 12 | 100.0 | 0 | 0.0 |
| Difficulty sleeping | 10 | 0.5 | 10 | 100.0 | 0 | 0.0 |
| Itching | 9 | 0.5 | 9 | 100.0 | 0 | 0.0 |
| Abdominal distension | 5 | 0.3 | 5 | 100.0 | 0 | 0.0 |
| Hypertension | 3 | 0.2 | 3 | 100.0 | 0 | 0.0 |
| Difficulty hearing | 3 | 0.2 | 3 | 100.0 | 0 | 0.0 |
| Swollen eye | 1 | 0.1 | 1 | 100.0 | 0 | 0.0 |
| Red eye | 1 | 0.1 | 1 | 100.0 | 0 | 0.0 |
| Swollen face | 1 | 0.1 | 0 | 0.0 | 1 | 100 |
| Burning micturition | 1 | 0.1 | 1 | 100.0 | 0 | 0.0 |
| Toothache | 1 | 0.1 | 1 | 100.0 | 0 | 0.0 |

An adverse event defined as when a participant complained of a symptom at any time point during drug administration and day 7 follow up. Among total of 1999 participants who received MDA, 909 (45.5%) complained of AE.

# Percentage of AE = An AE / Total number of participants received scheduled MDA*100

*Severity= Number pf participants with particular grading / Number of participants with corresponding AE.

Severity graded as mild when a participant did not sought treatment at any time-point of a particular AE and severity graded as moderate when a participant sought treatment at any time-point of a particular AE
