# Supplementary material for: A Controlled Trial of Mass Drug Administration to Interrupt Transmission of Multidrug-Resistant Falciparum Malaria in Cambodian Villages
Source: Clin Infect Dis. 2018 Mar 7;67(6):817–26. doi: 10.1093/cid/ciy196 (PMC6117448; doi:10.1093/cid/ciy196)
Supplement: Supplementary Table 3 [file ciy196_suppl_supplementary-table-3.docx]

Supplement Table 3: Timeline, transmission season, uPCR surveys and MDA schedule.

|  | 2015 | | | | | | 2016 | | | | | | | | |
| --- | --- | --- | --- | --- | --- | --- | --- | --- | --- | --- | --- | --- | --- | --- | --- |
| Month | Jul | Aug | Sep | Oct | Nov | Dec | Jan | Feb | Mar | Apr | May | Jun | Jul | Aug | Sep |
| Malaria season | Main Transmission Season | | | | | | | Low  Season | | | Main Transmission Season | | | | |
| Survey | Month  0 |  | | Month  3 |  | | Month  6 |  | | Month  9 |  | | Month 12 |  | |
| MDA in intervention villages | 1st round | 2nd round | 3rd round | Treatment of new comers in intervention villages | | | | | | | | | |  | |
| MDA in Control villages | No drug administration | | | | | | | | | | | | 1st round | 2nd round | 3rd round |
